# Wild Bearded Capuchin Monkeys (*Sapajus libidinosus*) Strategically Place Nuts in a Stable Position during Nut-Cracking

**DOI:** 10.1371/journal.pone.0056182

**Published:** 2013-02-27

**Authors:** Dorothy M. Fragaszy, Qing Liu, Barth W. Wright, Angellica Allen, Callie Welch Brown, Elisabetta Visalberghi

**Affiliations:** 1 Department of Psychology, University of Georgia, Athens, Georgia, United States of America; 2 Department of Anatomy, Kansas City University of Medicine and Biosciences, Kansas City, Missouri, United States of America; 3 Unit of Cognitive Primatology, Institute of Science and Technology of Cognition, National Research Council, Rome, Italy; University of Florence, Italy

## Abstract

Humans can use hand tools smoothly and effectively in varying circumstances; in other words, skillfully. A few other species of primates crack encased foods using hammer tools and anvils. Are they skilled? Positioning the food on the anvil so that it does not fall off when struck is a component of skilled cracking. We discovered that bearded capuchin monkeys deliberately place palm nuts in a relatively stable position on the anvil before striking them. In the first experiment, we marked the meridians of palm nuts where they stopped when rolled on a flat surface (“Stop meridian”). We videotaped monkeys as they cracked these nuts on an anvil. In playback we coded the position of the Stop meridian prior to each strike. Monkeys typically knocked the nuts on the anvil a few times before releasing them in a pit. They positioned the nuts so that the Stop meridian was within 30 degrees of vertical with respect to gravity more often than expected, and the nuts rarely moved after the monkeys released them. In the second experiment, 14 blindfolded people (7 men) asked to position marked nuts on an anvil as if to crack them reliably placed them with the Stop meridian in the same position as the monkeys did. In the third experiment, two people judged that palm nuts are most bilaterally symmetric along a meridian on, or close to, the Stop meridian. Thus the monkeys reliably placed the more symmetrical side of the nuts against the side of the pit, and the nuts reliably remained stationary when released. Monkeys apparently used information gained from knocking the nut to achieve this position. Thus, monkeys place the nuts skillfully, strategically managing the fit between the variable nuts and pits in the anvil, and skilled placement depends upon information generated by manual action.

## Introduction

According to a dynamic view of human activity, skilled activity is evident when the user can achieve similar outcomes under variable conditions, smoothly and efficiently [1]. Skilled tool users achieve similar outcomes, smoothly and efficiently, when using different tools with different surfaces or components in the situation (e.g., using different scissors to cut different kinds of cloth, and cutting the intended pattern accurately in all conditions). Illuminating the range of situational factors that the tool user can manage strategically, and how she/he manages these factors, increases our understanding of the nature of skilled tool use. For example, a skilled stone knapper can create flakes of more uniform thickness and shape from variable stones, while controlling within a narrower range the kinetic force with which the striking stone hits the platform stone, and using less energy to do so, than less skilled knappers [2]. This approach to skilled activity highlights the embodied character of knowledge in using a tool, in accord with an embodied view of cognition [3] that eschews representational explanations of skilled behavior. One goal of our research program is to bring this theoretical approach to the study of tool use in nonhuman animals [4]–[6]. In this report, we consider actions by a nonhuman primate species, the bearded capuchin monkeys (*Sapajus libidinosus*, formerly known as *Cebus libidinosus*), that reveal strategic management of situational factors in a tool-using activity.

Bearded capuchin monkeys at several sites across their geographic range in the northeast of Brazil crack nuts on anvils using stones as hammers [7], and the monkeys at our study site, Fazenda Boa Vista (hereafter, FBV) do so habitually [8]. Our team has shown in previous studies that the monkeys in FBV select hammer stones by weight and material, nuts by resistance to cracking, and anvil sites by their relation to the efficiency of cracking and their recent use by others [4], [9], [10], [11]. Systematic selection of nuts, stones, and anvil sites implies attention to the various properties of these objects and surfaces with respect to their contribution to cracking nuts. It seems likely that the monkeys make these selections to optimize some combination of values (e.g., minimizing the time required to process a nut; maximizing the energetic return; maximizing the reliability of energetic gain, minimizing the risk of displacement or theft by group members). Here we report on behavioral management of another component of the nut-cracking cycle requiring accommodation to variable elements: placement of the nut onto the anvil prior to striking it.

We noticed that anvil sites have one or more pits and that the monkeys typically knock the nuts in pits on the anvils repetitively before releasing them in the pit and striking them with a stone. They use a rapid motion when knocking, raising the nut a few centimeters above the anvil and lowering the whole nut into the pit while holding it in one hand. We proposed that the monkeys perceive through knocking when the nut is in a stable position in the pit, so that it remains stationary upon release (which is necessary for effective cracking). In Study 1, we tested whether the monkeys systematically placed the most resistant palm nuts that they crack, piassava nuts (*Orbignya* spp.) in a particular orientation, if the monkeys released the nuts in a stable position, and if individual consistency in this aspect of behavior was related to efficiency of cracking. Piassava nuts require more strikes to open than other palm nuts in the area [11], and they are more resistant to fracture in laboratory testing [12]. Thus these nuts require the most effective handling to crack them efficiently. Effective handling includes releasing the nut in a stable position. To determine if the monkeys released the nuts in a stable position, we presented the monkeys individually with piassava nuts with meridians marked by us so that we could recognize on which side the nut stopped when rolled on a flat surface. Subsequently, in Study 2, we asked blindfolded human participants to place similarly marked piassava nuts into a pit as if to crack them, to determine if the behaviors we observed in the monkeys could reflect use of haptic perception to place the nuts, and to compare the behaviors with the nuts prior to placement used by the two species. In Study 3, to explore the physical properties of the nuts which informed positioning them by touch, we evaluated if the most bilaterally symmetric meridian of the piassava nuts, as judged visually by humans, corresponded with the meridian on which the nut stopped when rolled on a flat surface.

In sum, we explored the possibility that bearded capuchin monkeys strategically positioned irregularly contoured nuts on the anvil surface in such a way that they remained stable when released and when struck with a stone to crack them. We asked whether humans would do the same in the absence of vision. Finally, we asked whether the contours of the nuts could guide positioning. We confirmed that the monkeys do consistently place the nuts in a stable position, as do people, and that this position is associated systematically with the exterior contours of the irregularly shaped nuts. Specifically, monkeys and people preferentially place a rounder surface of the nut facing the sides of the pit in the anvil in which the nut is normally placed, and thus a flatter surface of the nut parallel to the anvil's top surface. People do this reliably when blindfolded, indicating that kinesthetic or haptic cues are sufficient to guide this preference. However, monkeys and people use different exploratory actions, *sensu* Lederman and Klatzky [13], to discover the contours of the nuts and to detect when the nut is in a stable position on the anvil. Humans rotate the nut in the hand and sometimes also in the pit of the anvil; monkeys knock the nut against the anvil surface. To manage this particular challenge each species makes use of species-typical manual actions used routinely in many other exploratory situations.

## Study 1. Monkeys Positioning Nuts

### Methods

#### Subjects and site

Our site is located at Fazenda Boa Vista and adjacent lands (hereafter, FBV) in the southern Parnaíba Basin (9°39′S, 45°25′W) in Piauí, Brazil. Boa Vista is a flat open woodland (altitude 420 m asl) punctuated by sandstone ridges, pinnacles and mesas rising steeply to 20–100 m above the plain. A more complete description of the site can be found at http://www.EthoCebus.net and Spagnoletti et al. [8].

Ten monkeys (8 males and 2 females; 4 juveniles, 2–3.5 years old, and 6 adults) in one group of wild bearded capuchin monkeys participated voluntarily. The monkeys are habituated to close human presence. They come regularly to a flat, wooded area about 1500 m^2^ containing several natural anvils (boulders and logs) and hammer stones, and we use this area as our field laboratory. We conducted the study in June 2009 over the course of two weeks.

#### Materials

**Figure 1:**
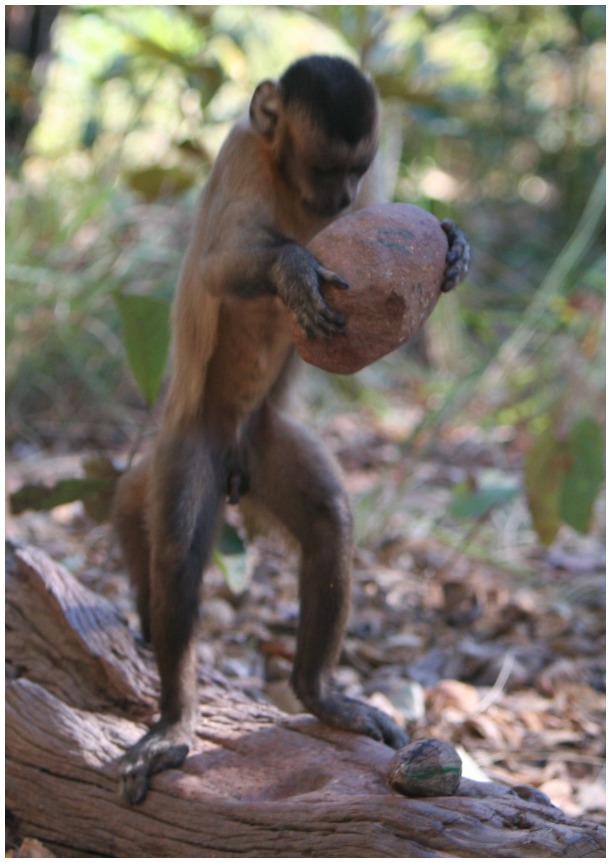
Wild bearded capuchin monkeys place nuts in a stable position on an anvil before striking them with a stone to crack them. The black line on the nut shown in this photograph (lower right) marks where the nut stopped when rolled on a flat surface (the Stop meridian). The green line shows the meridian at 90° from the Stop meridian (the Roll meridian). The monkeys consistently placed nuts marked in this way with the Stop meridian facing vertically, as shown in the photograph. Photo by B. Wright.

We presented the monkeys with familiar quartzite stones, weighing 930 g, 1113 g or 1460 g, placed on or next to a log anvil used by all the monkeys also outside of experimental sessions (see Figure 1). The anvil contained two pits produced by monkeys striking nuts (1.9 cm and 1.0 cm deep), and flat surfaces on either side of the pits.

**Figure 2:**
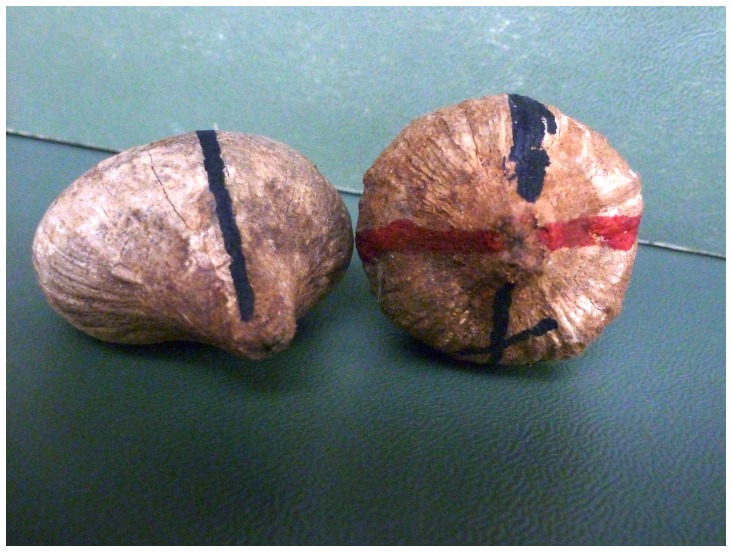
Position of the nuts on the anvil was assessed using visible markings. Marked nuts (piassava, *Orbignya* spp.) showing the Stop meridian on each nut (solid lines) and Roll meridian (red line on the nut on the right). The two nuts illustrate the variability in the shape of these nuts. Photo by D. Fragaszy.

**Figure 3:**
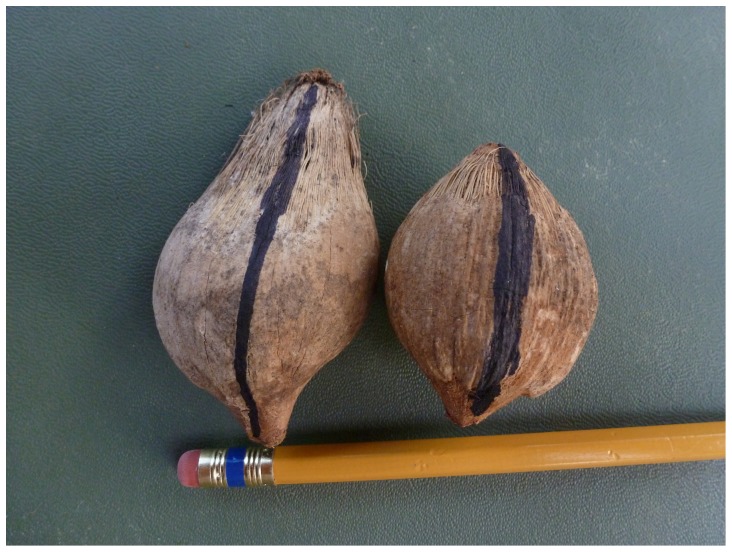
The same nuts as shown in Figure 2, here seen from above, showing the Stop meridian. Photo by D. Fragaszy.

We provided local palm nuts (*Orbignya* spp.), that on average are 60 mm long and 41 mm diameter and ellipsoid in shape [12] (see Figures 2 and 3). The mesocarp of the nuts was removed, as the monkeys remove the mesocarp before cracking them. To determine the flatter side of each nut, we rolled the nut on a flat concrete floor. When the nut came to a stop, we marked a straight line on the upper surface and a black cross-hatch pattern (a straight line with slashes through it) on the surface against the floor (a longitudinal meridian; the Stop Meridian) using a black marker pen. We marked a second longitudinal meridian at 90 degrees (Roll Meridian) in red or green. Two marked nuts are shown in Figures 2 and 3. We numbered each nut with a marker pen.

#### Procedure

After the stones were placed by the anvil, a marked nut was placed near the anvil when the monkeys were nearby. A trial began when a monkey approached the anvil and started manipulating the nut and a stone and ended when the subject finished cracking (successfully or unsuccessfully). We filmed all trials using a Canon GL2 digital video camera, recording at 30 fps, from a distance of 6–8 m with close focus on the hand, nut and anvil. The observer narrated the identity of the monkey, the number of the nut, which meridian faced up (within 30° of vertical) when the monkey released it and prior to each strike, as possible, and if the nut was cracked (see Video S1). We filmed in accord with the monkeys' interest in the task, obtaining from 3 to 106 strikes per monkey (Median = 15.5 strikes) over 3 days.

The tapes were coded twice using Observer (versions 5 and XT; Noldus Corporation) in slow-motion and stop-action playback. First, for each trial, the position of the nut on the anvil (i.e., the position of the Meridian lines) per strike, whether one or two hands were used to place the nut, movements of the nut (wobbles or rolls) following placement, the number of strikes directed at that nut, and the outcome (nut cracked or not) were coded for the six adults and two 3.5-year-old juveniles producing 10 or more strikes each. The position of the Meridian lines was coded in two ways: by determining (1) if the Stop Meridian was within 30° of the vertical, and if so, (2) which line, hatched or straight, was facing up. A wobble was defined as visible movement of the nut but it remained in one place; a roll was defined as movement of the nut away from where the monkey positioned it. Following training with an experienced coder (DF), AA conducted this coding, consulting with DF in cases of ambiguity. The few ambiguities were resolved by the two coders reviewing together the episodes in question.

The tapes were coded a second time for all 10 monkeys for manual actions preceding each strike (see Table 1). Taps were defined as light, repetitive percussion with the finger tips, typically of a nut held in the other hand; knocks were defined as forceful percussion of the nut against a hard surface, accompanied by an audible signal, and the nut was not released in the same motion. We noted against which surface the nut was knocked. Reliability was established for this coding by two independent coders (DF and CWB) coding 5% of the data set, achieving 100% agreement on frequency of strikes and knock events and the location of knocks. CW coded the data. The episodes with the best visibility of these events were coded in this way; this constituted 55% of the data set coded for position of the nuts in the first pass through the video.

**Table 1:** Actions preceding each strike.

Variable	Definition
Tap	Taps fingers on nut
Strike	Monkey strikes the nut on the anvil with the stone hammer
Release	Full release of nut from hand(s); visible space between hand(s) and nut
Knock on pit	Nut is held in hand and struck against a pit in the anvil
Knock on rim	Nut is held in hand and struck on the rim of a pit (the boundary between the flat surface and the pit)
Knock on stone	Nut is held in hand and struck against the stone hammer
Knock on flat	Nut is held in hand and struck against the anvil on a flat area (not the pit or rim)
Knock off camera	Behavior is not on camera or not visible to viewer, but sound suggests the nut is struck against some surface
Rotate in the pit	Nut is rotated around a center point so that the long axis points in a different direction upon release.
Manual rock	Nut is pushed on one end as it rests in pit so that one end is raised and then lowered.
Rotate while held above the anvil	Monkey moves the whole nut a few degrees while holding it above the pit by flexing the wrist, then replaces it in the pit

This study was approved by the IACUC of the University of Georgia (AUP # 2010 04-067-Y3-A0 and # 2009 02-035-Y3-A0) and conducted in accord with all relevant local and national regulations regarding the humane use of animals in research.

#### Analysis

We tabulated the data by individual and evaluated per subject, for the eight subjects that produced 10 or more strikes, the probability that the Stop Meridian faced up more often than expected by chance using Chi Square tests. For those subjects that produced 10 or more events in which the Stop Meridian faced up, we evaluated the distribution of the straight line or cross-hatch line facing up, also using Chi Square tests. We derived from the tabulated data, per individual, efficiency (defined as average # strikes to open a nut, calculated as # strikes/# nuts cracked), proportion of placements with two hands, rate of knocks and taps per strike, movements of the nut in or above the pit (rotation, rocking) before release, and frequency of wobbles and rolls following release.

We correlated efficiency with percent of placements with the Stop Meridian facing up using the Spearman correlation (N = 8). For all statistical tests we used a two-tailed alpha = .05.

### Results

All 8 monkeys producing 10 or more strikes reliably put the nut into the pit with the Stop Meridian 1 facing up (*X^2^* = 9.31 to 36.26, 1 df, all p<.05). Overall, there were 253 placements with the Stop Meridian facing up out of 302 placements that could be coded for this variable (84% of coded placements). Twenty-eight placements could not be coded for this variable because the camera view of the nut was occluded. Variability across monkeys in the proportion of placements with the Stop Meridian facing up was rather low; individual values ranged from 0.71 to 0.94 (Med = .81). In 45% of placements, the solid line of Meridian 1 faced up, and in 39% of placements, the hatched line faced up (difference NS for every subject).

Individual efficiency (average # strikes to open the nut) ranged from 1.0 (optimal) to 16.0 (Med = 5.0). The Spearman correlation between efficiency and % placements with the Stop Meridian facing up was −0.75 (N = 7 individuals that opened a nut, p<.10, 2 -tailed). Bimanual actions to place the nut were rare (6% of placements).

The data for actions with the nut preceding each strike for 10 monkeys are shown in Table 2. Of 558 Knocks coded, 15 happened off camera (i.e., we heard the knock in the audio track but could not specify where the nut was knocked). Typically the monkeys knocked the nut in the pit repetitively before releasing it (Median = 5 knocks per strike, IQR = 1.3–6.9). Of the 543 knocks that were visible, the monkeys knocked the nut most often in the pit (31%) or on the rim of the pit (41%). They knocked the nut on the stone on 16% of Knocks, and the remainder on a flat part of the anvil. The monkeys rarely tapped the nut with their fingertips (median = 1); no monkey performed this action more than 3 times. The nut wobbled after the monkey released it just 10 times (out of 253 placements coded for this variable), across 4 individuals (range 1–5 times), and the nut rolled after release just once, during a placement by a juvenile. Monkeys rarely rotated the nut in the pit or on the flat surface of the anvil before releasing it (14 times out of 253 placements coded for this variable). They never adjusted the position of the nut in the air above the anvil, nor felt the pit with their hands, except to sweep away debris.

**Table 2:** Actions made by monkeys with the nut preceding each strike.

	Tap	Knock in pit	Knock on rim*	Knock nut on stone	Knock on flat#	Knock out of camera view	Total Knocks	Strikes	Ratio Knocks: Strikes
Catu	0	3	0	4	5	0	12	18	1.67
Chuchu	1	46	5	10	2	0	63	21	3.00
Dengoso	1	6	15	35	16	4	76	11	6.91
Jatoba	1	14	12	1	2	0	29	23	1.26
Mansinho	0	9	0	0	0	0	9	25	0.36
Pati	3	12	4	11	9	0	36	14	2.57
Teimoso	1	32	6	0	1	7	46	27	1.70
Tomate	0	31	4	17	1	0	35	17	2.06
Tucum	0	14	4	5	9	3	35	21	1.67
Caboclo	3	4	4	3	16	1	28	9	3.11
MEAN							36.9	18.6	1.98

* Rim of the pit in the anvil's surface.

# Flat surface on the anvil away from the pit.

### Discussion

We examined whether bearded capuchin monkeys placed nuts into a pit on an anvil in a particular orientation before striking them with a hammer stone, and if so, if this placement was related to efficiency of cracking. All eight monkeys placed the nuts systematically (78% or more of placements) in such a way that the Stop meridian, the meridian along which the nut stopped rolling on a flat floor, was facing within 30 degrees of vertical with respect to gravity (whereas random placement would produce this result 17% of the time). There was directional variation in the proportional frequency of placing the Stop meridian facing up, with a trend for more efficient monkeys (using fewer strikes to open a nut) to do so more often. However, the relatively high and consistent values for this variable across individuals (spanning a range of ages and skill at cracking) indicates that the monkeys are attentive to this aspect of the cracking action, even when they are not yet skillful.

The Stop meridian facing up was a stable position for the nuts, as indicated by the near absence of wobbling or rolling following release. Overall, the consistency of placement and the lack of movement after placement implies that the monkeys determined when the nut was in a stable position prior to releasing it, and they did so even when not skillful at cracking.

The monkeys typically employ a distinctive behavior, knocking the nuts on the anvil, prior to placement. Knocking likely provides the monkeys with information about the fit between the nut and the pit, perhaps from the sound and/or perhaps from the vibrations of the nut after it strikes the anvil surface. We have data from other contexts indicating that exploratory manual actions inform the monkeys' selection of stones to use as hammers, when more than one stone is near the anvil. The monkeys frequently touch and tap stones with their fingers before choosing one to use for cracking [9], [11]. They appear to use exploratory actions to choose pits as well [4], moving after one or a few positioning events and/or strikes from pits in which they have lower efficiency at cracking to pits where they have higher efficiency. Taken together, these findings suggest that the capuchins make substantial use of haptic and/or auditory perception in nut cracking, including perception of the relation between nut and pit and among nut, pit and stone when striking the nut.

Although many aspects of haptic perception in humans have been characterized [14], the sensory basis for perception of stability with respect to gravity of a hard object struck against a hard surface is not among them. Our suspicion is that frequency and magnitude of vibration (of the nut, in this case) inform this perception. Fast-acting mechanoreceptors in the hands of primates (Meissner's corpuscles, densely present in the finger tips, which are maximally sensitive to temporal frequencies of vibrations between 3 and 40 HZ, and Pacinian corpuscles, located most densely in the palm, which are particularly sensitive to vibration from 150 to 300 Hz) [15] could provide the necessary sensory acuity for this perception. The density of Meissner's corpuscles in the digits of five species of nonhuman primates examined by Hoffman et al. [16] ranged from 7.2 to 44.6 per mm^−2^, which encompasses the value reported for Digit 1 in humans (16–17 per mm^−2^). Thus nonhuman primates probably have equivalent or even enhanced perception of surface properties sensed by these receptors, compared to humans. Equivalent information is not yet available for Pacinian corpuscles. The density of Meissner's corpuscles correlated positively with the extent of frugivory in the species examined by Hoffman et al. [16], and the authors suggest that digital sensitivity conferred by these receptors, and reflected in the somatosensory portions of the brain (elaborated in primates compared to other mammals [17]) function to enhance exploration and handling of objects in foraging. The dexterous and varied repertoire of manual actions used by capuchins in foraging suggests that they use their hands for exploration of texture, firmness, and other object properties, using active touch [18]. Perhaps they can use haptic tactile perception to judge the fit between nut and anvil surface.

Acoustic information could also inform perceivers about the relations between objects and surfaces. People can accurately specify many object properties by sound alone, which reflects the fact that “sound is structured reliably by interacting materials” ([19], p. 4). It seems probable that capuchin monkeys can do the same, given the similarities in auditory sensitivity across primates [20], [21].

Humans also use active haptic sensing touch to gain fundamental information about objects and surfaces [13], [14], [22]. Dynamic touch is an integral part of goal-directed action in humans, including selecting and using objects as tools, from a young age. For example, children as young as four years old can accurately judge by manual action the rigidity of an object they will use to mix a cup of sugar [23]. Nevertheless, humans preferentially rely on vision when possible, from the youngest ages, to select and to grasp objects for various purposes [23], [24], [25]. It may be that capuchin monkeys rely on haptic and/or auditory perception more than humans in situations where humans rely mostly on vision (such as positioning an object held in the hand into a pit).

One reason why capuchin monkeys may not rely on vision as much as humans may be that wild capuchin monkeys are particularly visually vigilant, glancing around themselves and away from the task at hand, and they do so more often when they crack nuts than during other activities [26]. Thus they may be looking at their surroundings rather than at the nut and pit while positioning the nut.

One conclusion we can draw from these findings is that a different profile of reliance on particular senses in skilled activity could be an important source of differences between capuchins and humans in how they approach a tool-using problem. A problem that can be evaluated primarily visually by humans may not be so evaluated by capuchin monkeys. Therefore, experiments that aim to examine tool-using abilities in non-human animals, even primates, need to take the species' unique perceptual profile into consideration. We cannot assume that a particular task would be evaluated by the animals in the same way that we would evaluate it (i.e. by visual inspection).

## Study 2. Humans Positioning Nuts

The findings of Study 1 indicate that bearded capuchin monkeys, prior to cracking nuts, systematically placed them into pits on an anvil with the Stop meridian facing upward (i.e., vertically with respect to gravity). They typically knock the nuts on the anvil repetitively prior to placement. We interpreted this as evidence that the monkeys perceive the stability of the nut's position in the pit using active haptic sensing (*sensu* 14). Active haptic sensing occurs when the hand moves voluntarily over a surface or object. This mode of activity has an exploratory character and it is the usual and preferred activity for humans identifying objects and extracting information about them.

To determine if humans also position nuts systematically using active haptic sensing, as partial confirmation of our interpretation of the monkeys' behavior, we asked human participants, while blindfolded, to place nuts into the pit of an anvil as they would to crack them.

### Methods

#### Subjects

Seven women (ages 16–58) and seven men (ages 14–33) participated. We conducted the study at the field laboratory at FBV. Each person was blindfolded during testing. Written informed consent was obtained from each participant, and in the case of minors, from the parent as well.

#### Materials

We used a log anvil used by the monkeys that contained two natural pits formed by monkeys striking nuts and flat surfaces on either side of each pit. This anvil was very similar to the one used by the monkeys in Experiment 1. We presented 20 palm nuts (piassava; *Orbignya* spp.), on average 60 mm long and 41 mm diameter, as in Study 1 (see Figure 2). Each nut was marked along the Stop meridian in same manner as in Experiment 1. We recorded all trials using a video camera (Canon GL2) recording at 30 fps.

Participants sat on a low stool at a distance that afforded a comfortable reach to the anvil. A cloth scarf was used as a blindfold.

#### Procedure

Each person was requested to position nuts during a test session lasting about 5 minutes. The participant was instructed on the procedure, shown the anvil, and asked to position himself/herself comfortably to reach the anvil from the seated position, and blindfolded. The experimenter handed each of the 20 nuts, one at a time, to the participant. Participants were encouraged to feel the anvil and to handle the nut as they liked, for as long as they liked, and then, using one hand, to place the nut on the anvil as they would if they were preparing to crack it using a stone. Another experimenter recorded the position of the nut on the anvil and whether the nut wobbled or rolled upon release (thus these variables were coded in real time). On two trials per participant for participants 1–9, a third person provided an independent judgment of the nut's position. These data were used to calculate inter-observer reliability for the judgment of nut position. Inter-observer agreement (calculated as [agree/agree+disagree]×100) was determined to be better than 90% for all variables coded.

From video recording, we coded the same variables as coded for the monkeys: position of the stop meridian (within 30° of vertical, or not), which line was up (hatched or straight), whether the nut wobbled or rolled upon release, and all occurrences of each person's actions with each nut prior to placement using the same ethogram used for the monkeys (see Table 1). This list encompasses all the forms of unimanual actions people used with the nuts in the course of placing them on the anvil.

In addition to presenting descriptive results of people's performance, we compared the proportion of nuts placed with the Stop meridian facing up and the frequency of the various actions with the nuts by humans and by capuchin monkeys using the Mann-Whitney test, with two-tailed alpha = .05.

The procedure was approved by the Institutional Review Board of the University of Georgia (#2006-10469-4 and #2013-10236-0).

### Results

Humans, like monkeys, usually positioned the nut with the Stop meridian facing the anvil and upward. They positioned the Stop meridian within 30 degrees of vertical orientation on average 15.6 times (71%; Range = 11/20 to 19/20) out of 20 placements. This outcome is similar to the monkeys' data: monkeys' values ranged from 71% to 94%. The hatched line faced up on 41% of trials, indicating that humans, like the monkeys, did not distinguish between the “top” or “bottom” of the Stop Meridian. Monkeys placed the hatched line facing up on 39% of placements.

The humans placed the nuts with similar stability as the monkeys (4 wobbles per 280 placements vs. 10 in 330 placements, and 3 rolls for humans vs. 1 for the monkeys). Four people (out of 14) produced rolls or wobbles. However, humans did not often knock the nuts in the pits before releasing them, as the monkeys did, but instead used actions that the monkeys did not (pressing on the nut after releasing it, for example). Humans often rotated the nut in the pit on average once per nut, and rolled the nut using the finger tips on average once per nut. One person rocked the nut twice by pushing on one end of the nut as it rested in the pit. Three individuals knocked the nut in the pit a total of 13 times (out of 100 placements for N = 5). Humans often felt the pit with their fingers before placing the nut in it, and handled the nut above the anvil using in-hand movements prior to contacting the anvil. In comparison, the monkeys rotated the nut occasionally (14 times out of 330 placements), but never felt the pits with their hands except to clean debris out of them with a quick sweeping action. Monkeys never moved the nut with in-hand movements. However, they typically knocked the nut against the anvil or the stone several times before each strike.

### Discussion

Like the monkeys, humans reliably placed the nut into the pit in the anvil with the Stop meridian (the meridian on which the nut stopped when rolled on a flat floor) facing within 30 degrees of vertical when placing the nut without using vision. We observed greater variation in this behavior across humans than across monkeys, suggesting more variable attention to haptic cues by humans unfamiliar with this particular task than by the more experienced monkeys. Humans used different manual actions than the monkeys to position the nuts. Humans rotated the nut using the fingers in the hand holding the nut (in-hand movements [27]), and the capuchins did not. Instead, the monkeys struck the nut repetitively against the surface of the pit prior to placement, whereas humans rarely used this action. Of course, humans have larger hands than the monkeys and perhaps this difference explains some of the differences in how each species handled the nuts.

Nevertheless, the end result was the same: Both monkeys and humans, the latter inexperienced at the task and blindfolded, usually released the nuts in a stable position, with the Stop meridian facing vertically (into the pit) and the Roll meridian positioned horizontally (facing the edges of the pit). This finding provides convergent support for the hypothesis that the bearded capuchin monkeys use haptic cues to position the nuts.

It seems plausible that some feature of the nuts makes orienting the Stop meridian into the pit a more stable position or provides some other advantage in cracking the nut. In the next study we examine a feature of the nuts that may produce such an advantage.

## Study 3. Relation between Stop and Roll Meridians and Nut Contours

Given that both monkeys and humans (while the latter are blindfolded) systematically placed the Roll meridian more horizontal than the Stop meridian, there is some property of these meridians, and/or some relation between these meridians and the pits in the anvil, which is accessible to touch and which is guiding placement. Piassava nuts vary in contour, in part in accord with the number of locules in the nut, which vary from one to six. Most nuts are asymmetrical in cross-section through the long axis of the nut. However, bilateral symmetry may still be present in one or more cross-sections; i.e., along one or more meridians (see Figures 2 and 3). We explored whether the Stop and Roll meridians had an orderly relationship with the occurrence of bilateral asymmetry along the long axis of the nuts. We did so in two phases. In the first phase, two people judged the most symmetrical meridian for 30 nuts already marked for Stop meridians. In the second phase, two other people judged the most symmetrical orientation of 40 unmarked nuts, and subsequently, a third person marked the Stop meridians in the same manner as for Studies 1 and 2, and a fourth person judged the angle between the Stop and “symmetry” meridians. In both phases, we evaluated the number of nuts for which the “symmetry” meridian and the Stop meridian were within 10° degrees, a more conservative rule than was used to evaluate verticality of the meridian with respect to gravity in Studies 1 and 2, and within 30°, the looser rule used to judge orientation of the nut in Studies 1 and 2.

### Phase 1

#### Materials

We used 30 piassava nuts, numbered and marked for Stop meridians as in Experiments 1 and 2. Using a cloth tape marked in millimeters, we measured the circumference of each nut on the Stop meridian and the Roll meridian (i.e., 90 degrees offset from the Stop meridian), wrapping the tape tightly along the contours of the nut. We determined that the circumference of the nuts about the Stop and Roll meridians did not differ systematically, and the absolute differences were small. The average circumference of the Stop meridians was 15.54 cm; for the Roll meridians, 15.60 cm. The largest difference in circumference of the two meridians for any nut was 0.9 cm.

#### Participants

Two adults, naïve to this study but familiar with piassava nuts, participated.

#### Procedure

Participants were asked to look at each of 30 marked piassava nuts and to indicate the meridian about the long axis of the nut where it appeared the most bilaterally symmetrical. We then noted the difference (in degrees) of the meridian they indicated with the Stop meridian.

The procedure was approved by the Institutional Review Board of the University of Georgia (#2006-10469-4 and #2013-10236-0). Written informed consent was obtained from each participant.

#### Results

Both participants indicated that the most bilaterally symmetrical orientation of the nut about its long axis occurred principally along the Stop meridian. One participant indicated the Stop meridian for 28 out of 30 nuts. In the remaining two cases, this participant indicated the Roll meridian. The second participant indicated the Stop meridian 24 times, a meridian within 30 degrees of the Stop meridian four times, and the Roll meridian twice. The two participants agreed for one nut that the most symmetrical meridian was the Roll meridian. Inspection of this nut revealed that it was nearly actinomorphic (radially symmetrical). Both participants indicated that the side of the Stop meridian facing the floor was the most symmetric side more often than the side facing upward when the nut rolled to a stop (26/30 and 21/30; X^2^(df = 1) = 16.13, p<.005, and 4.8, p<.05, respectively).

### Phase 2

#### Materials and Procedure

We used 40 randomly selected piassava nuts, red and black permanent marker pens, a protractor, and pencil and paper.

Two people visually judged and marked the symmetry meridian of 20 nuts each using a red marker pen. An experimenter determined the stop meridians and marked them in black permanent ink. A second experimenter judged the angle between the two marked meridians using the protractor and angles of 10 and 30 degrees drawn on paper as visual aids.

The procedure was approved by the Institutional Review Board of the University of Georgia (#2006-10469-4 and 2013-10236-0). Written informed consent was obtained from participants.

#### Results

For 21 nuts, the Stop meridian and the symmetry meridian were judged to fall within 10° of each other, and for 33 nuts these meridians were judged to be within 30°. For the first case, a 10° distance is expected on 1/8 cases, or 5 times for 40 nuts. The observed distribution of 21 nuts with these meridians within 10° of each other is significantly different than expected by chance (X^2^ (1) = 58.51; p<.001. For the second case, the expected distribution is one case out of three within 30°. The observed distribution, 33/40, produces a X^2^ of 43.67, p<.001.

#### Discussion

Given that the nuts have asymmetrical meridians in degree of flatness, but equivalent circumferences, the Roll meridian should be more symmetric in curvature than the Stop meridian, as illustrated in Figures 2 and 3. In Phase 1, two human participants confirmed this prediction, systematically indicating that the Stop meridian defined the most bilaterally symmetrical orientation of the nut about its long axis. In Phase 2, we replicated this result with a new sample of nuts and a slightly different procedure (the Stop meridian was marked after the symmetry judgment was made rather than before).

By placing the Stop meridian into the pit, the individual ensures that the most symmetrical contour of the nut faces the sides of the pit. This position probably provides the most contact between the nut and the sides of the pit, maximizing the stability of the nut's position during cracking. Thus we interpret this position as useful with respect to maintaining control of the nut during the cracking process. It may also have implications for the amount of force needed to crack the nut. The force of the strike is directed downward and not deflected sideways if the sides of the nut are confined when the nut is struck.

## General Discussion

A skilled individual uses minimal effort for maximum effect at the activity in question, and adjusts actions to accommodate minor variations in circumstances while producing a uniform result, among other features of skill [1], [2]. Adult wild bearded capuchin monkeys exhibit skill in several dimensions of nut-cracking, including selecting the nuts that are easier to crack [11], selecting anvil surfaces that support more efficient cracking [4], and selecting stones of the more appropriate size and composition [9], [11]. They also adjust the velocity and maximum displacement of the stone in response to the weight of the stone and the weight of the nut [28].

In Experiment 1 we showed that adult wild bearded capuchins monkeys use another action strategy that we propose is related to skill: they position the nut systematically in the pit before striking it. In Experiment 2, we showed that humans do the same when placing the nuts without vision, suggesting that haptic perception is sufficient for this behavior. In Experiment 3, we found that the strategy that both humans and monkeys use to place the nuts results in the most symmetric sides of the nut facing the walls of the pit and the more asymmetric sides facing vertically in the pit. We interpret this positioning as producing the most secure position of the nut with respect to movement during and following striking in accord with greater friction between the wall of the pit and sides of the nut in this position than in others.

Capuchins apparently detect the relevant properties of the nut they will place in the pit on the anvil by repetitively knocking the nut against a hard surface (the stone or the anvil, especially in or near the rim) before releasing it. All monkeys knocked the nut into the pit repetitively two or more times almost every time they position a nut, even when re-positioning a nut they have just struck. They do not handle the nuts in other ways that could generate perceptions of contour, for example by rolling the nut in their hands. Knocking (also called banging in the literature) is a species-typical action that appears early in life and is used ubiquitously by capuchins in foraging and more generally to explore objects [29]. Thus they have life-long experience learning about objects from this action.

Humans accomplished the same goal (placing the more asymmetric meridian facing vertically) with other actions than the monkeys use. Specifically, humans used species-typical in-hand movements [27] directly on the nut, such as rolling and rotating the nut in one hand, prior to bringing the nut into contact with the anvil. Humans also commonly used actions with the nut in contact with the pit, such as rotating, rolling and rocking the nut in the pit prior to releasing the nut. Perhaps these actions generate information about friction of nut with respect to the wall of the pit or the position of the center of mass with respect to gravity. Despite these differences in forms of manual action, both species released the nut in a stable position. The nuts rarely wobbled or rolled when humans or monkeys released them.

An important conclusion from the current studies, and others from our work at FBV, is that bearded capuchin monkeys' effective nut-cracking involves concurrent attention to several perceptual features of the problem and effective modulation of activity in accord with variable circumstances. This behavior is “skilled” in Bernstein's sense of the word [1]. This conclusion leads to some specific predictions that we aim to test. For example, we predict that young monkeys learning to crack piassava nuts will position nuts with the more symmetrical sides against the walls of the pit proportionally less often than more skilled individuals, and/or that when they release them the nuts will roll or wobble more often than those positioned by more skilled nut-crackers.

We suggest that attention to the perception/action features of skilled behaviors, in accord with an embodied approach to cognition [3], will enrich our understanding of varied forms of tool use (using rakes, probes, and containers, for example) and indeed, problem-solving behaviors in general, particularly in nonhuman species [30]. An embodied approach to skilled activity [31] leads us to ask what features of a problem constitute affordances for action for the individuals involved, and what constitutes information guiding individuals' goal-directed actions in given circumstances. In the particular case of placing nuts into pits on anvils before striking them with a stone, both monkeys and humans position nuts in a certain way in the pit, but they use different actions to achieve this outcome. In the language of embodied cognition, the two species perceive the same affordances, although they use different sources of information, and different actions, to do so. Much of the work in embodied cognition about defining affordances and determining sources of information available through perception concerns vision [31]. We show here that haptic perception also provides fertile ground for study from this perspective.

## Supporting Information

Video S1 This video shows two episodes in which a bearded capuchin monkey places and strikes a nut with the Stop meridian marked with a black line or a black-hatched line, and the Roll meridian marked with a red or green line.
(MP4)
